# Skeletal phenotype amelioration in mucopolysaccharidosis VI requires intervention at the earliest stages of postnatal development

**DOI:** 10.1172/jci.insight.171312

**Published:** 2023-11-08

**Authors:** Elizabeth Hwang-Wong, Gabrielle Amar, Nanditha Das, Xiaoli Zhang, Nina Aaron, Kirsten Gale, Nyanza Rothman, Massimo Fante, Andrew Baik, Ajay Bhargava, Arun Fricker, Michelle McAlister, Jeremy Rabinowitz, John Lees-Shepard, Kalyan Nannuru, Aris N. Economides, Katherine D. Cygnar

**Affiliations:** Regeneron Pharmaceuticals, Tarrytown, New York, USA.

**Keywords:** Bone Biology, Cartilage, Lysosomes, Monogenic diseases

## Abstract

Mucopolysaccharidosis VI (MPS VI) is a rare lysosomal disease arising from impaired function of the enzyme arylsulfatase B (ARSB). This impairment causes aberrant accumulation of dermatan sulfate, a glycosaminoglycan (GAG) abundant in cartilage. While clinical severity varies along with age at first symptom manifestation, MPS VI usually presents early and strongly affects the skeleton. Current enzyme replacement therapy (ERT) does not provide effective treatment for the skeletal manifestations of MPS VI. This lack of efficacy may be due to an inability of ERT to reach affected cells or to the irreversibility of the disease. To address the question of reversibility of skeletal phenotypes, we generated a conditional by inversion (COIN) mouse model of MPS VI, *Arsb^COIN/COIN^*, wherein *Arsb* is initially null and can be restored to WT using Cre. We restored *Arsb* at different times during postnatal development, using a tamoxifen-dependent global Cre driver. By restoring *Arsb* at P7, P21, and P56–P70, we determined that skeletal phenotypes can be fully rescued if *Arsb* restoration occurs at P7, while only achieving partial rescue at P21 and no significant rescue at P56–P70. This work has highlighted the importance of early intervention in patients with MPS VI to maximize therapeutic impact.

## Introduction

Mucopolysaccharidoses (MPS) are a subset of lysosomal diseases that are caused by a recessive deficiency in enzymes required for the degradation of glycosaminoglycans (GAGs) (1–3). GAGs are functionally diverse but play critical roles in tissue maturation, extracellular matrix (ECM) formation and function, and the development of the skeleton. Seven MPS subtypes have been described, and although genetically distinct, they display substantial overlap in clinical phenotypes. Nearly all MPS subtypes include a spectrum of dysostosis multiplex, also referred to as progressive skeletal dysplasia (1–3).

Mucopolysaccharidosis VI (MPS VI), otherwise known as Maroteaux-Lamy syndrome (OMIM #253200), results from complete or partial loss of function of arylsulfatase B (*ARSB*) (4). ARSB is a lysosomal enzyme required for the degradation of dermatan sulfate (DS), also known as chondroitin sulfate B (4–7). ARSB loss causes accumulation of substrate in lysosomes, with downstream effects linked to lysosomal dysfunction, such as impaired vesicular trafficking, autophagy, and ion homeostasis, which likely contribute to clinical manifestations (8, 9).

Enzyme replacement therapy (ERT) using recombinant human ARSB (galsulfase) has been approved and available for chronic treatment of MPS VI since 2005 (10). In patients with MPS VI, galsulfase ERT quickly and effectively reduces urinary GAG and hepatosplenomegaly, demonstrating efficacy in treating soft-tissue manifestations. However, it achieves limited efficacy for the spectrum of skeletal manifestations, with a sporadically reported, modest improvement in shoulder flexion (2, 4, 11).

Previously studied animal models include naturally occurring MPS VI cats (OMIA:000666-9685), which exhibit facial dysmorphia, elevated urinary GAGs, osteopenia, impaired longitudinal tibial growth, and abnormal vertebral bone growth. Increases in growth plate width, particularly in resting and hypertrophic zones, provided evidence that the endochondral ossification process was affected with the loss of ARSB (12, 13). In MPS VI cats, galsulfase ERT failed to substantially effect most bone length parameters, although bone volume and trabecular volume readouts were improved with high doses. In addition, genetically engineered mouse models have been developed modeling specific *ARSB* mutations (11, 14, 15). They all share the same skeletal abnormalities, including osteopetrosis, decreased cranial length/width ratios, and decreased long bone length, and they demonstrate elevated GAG storage in visceral organs like the liver, heart, kidney, and spleen. These models have been used to explore efficacy of both traditional ERT and AAV delivered gene therapy (14, 15). Preceding the clinical experience with galsufase ERT, in these models, GAG level normalization could be achieved for visceral organs, yet both modalities failed to correct the dysostosis multiplex phenotype. Although a recent study exploring mandibular exostosis and retention of cuspids in both mouse and man suggests that better outcomes are observed with early delivery of ERT, the improved outcomes are limited to these 2 phenotypes (16). Hence, it is still unclear whether the lack of efficacy to correct the remaining skeletal phenotypes is due to poor uptake of recombinant enzyme in growth plate cells or whether established patterning defects and/or major cellular damage preclude skeletal rescue by the time of delivery.

In order to separate reversibility of phenotype from ERT delivery, we developed a new mouse model of MPS VI, employing a conditional inversion method (17). In this design, a region of murine *Arsb* encompassing exon 5 (ENSMUSE00001413545, Ensembl release 109) along with flanking intronic sequences is placed in the antisense orientation (inverted), rendering *Arsb* null. This region is also flanked by lox66 and lox71 sites in a mirror-image configuration to enable inversion by Cre. Therefore, when recombined by Cre, exon 5 (along with the flanking intronic sequences) is placed back to its native orientation, restoring *Arsb* to WT (Supplemental Figure 1; supplemental material available online with this article; https://doi.org/10.1172/jci.insight.171312DS1). Unlike classic floxed-stop conditional-on allele approaches, where WT ARSB might result from readthrough transcripts past a floxed-stop cassette (followed by RNA splicing to remove the cassette), in this conditional by inversion–based (COIN-based) approach, no functional message can be generated (17). We utilized this *Asrb^COIN^* allele together with a tamoxifen-controllable *Cre* recombinase, *Cre-ER^T2^*, to restore *Arsb* to WT at specific time points and, hence, achieve native ARSB expression from the endogenous locus at various developmental time points.

## Results

### Arsb COIN mice exhibit GAG accumulation and dysostosis multiplex.

**Figure 1:**
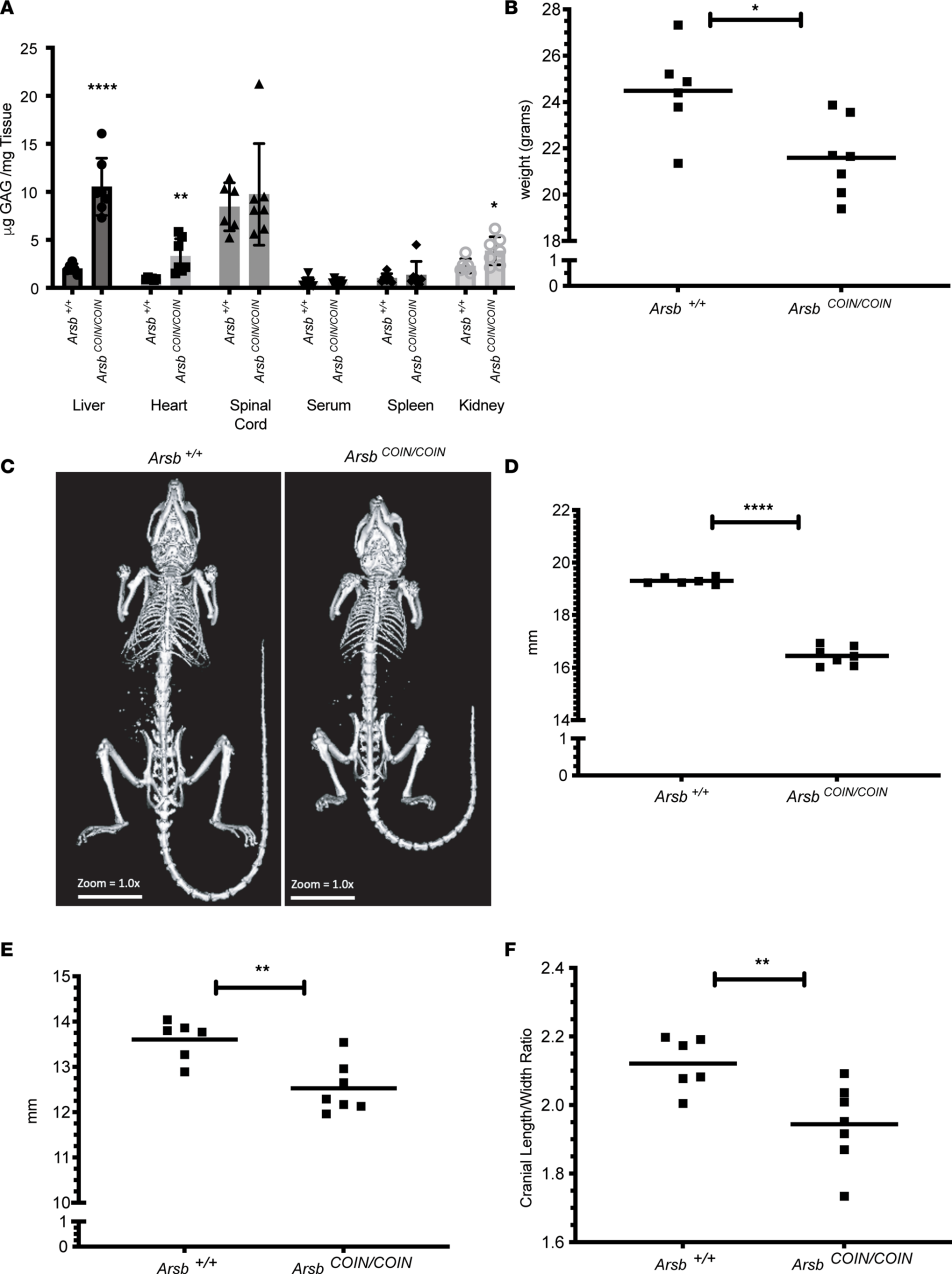
Arsb COIN mice replicate an MPS VI phenotype. (**A**) GAG accumulation in peripheral organs in 18- to 19-week-old females. (**B**) Total body weight of 18- to 19-week-old females. (**C**) Radiographs of 18- to 19-week-old WT versus Arsb COIN female mice. (**D**) Left tibia lengths in 18- to 19-week-old females. (**E**) L6 to L2 lengths in 18- to 19-week-old females. (**F**) Cranial length/width ratios in 18- to 19-week-old females. *n* = 6–7 mice. Statistical significance was determined by Welch’s *t* test. Data represent mean ± SD. *P < 0.05, **P < 0.01, ****P < 0.0001.

Since in *Arsb^COIN^*, exon 5 is placed in the antisense orientation, *Arsb^COIN/COIN^* mice start as functionally null. Nonetheless, we confirmed that *Arsb^COIN/COIN^* mice display key features of MPS VI (Figure 1, A–F). Eighteen- to 19-week-old female *Arsb^COIN/COIN^* mice were examined alongside an age- and sex-matched WT cohort for skeletal and peripheral tissue phenotypes. Total sulfated GAG in each tissue type was assessed using dimethylmethylene blue (DMMB), a chromogen that presents an absorption change upon binding the sulfated moiety in GAGs (Figure 1A) (18–20). Total sulfated GAGs were significantly elevated in liver, heart, and kidney of *Arsb^COIN/COIN^* mice (Figure 1A). In addition, these mice weighed significantly less (Figure 1B). Tomography revealed a difference in overall skeletal size (Figure 1C), with long bones (using tibia lengths as representative) of *Arsb^COIN/COIN^* mice remarkably shorter (Figure 1D). Comparison of vertebral column lengths (using L6–L2 measurements) also supported a significant skeletal phenotype (Figure 1E), as did cranial length/width ratios (Figure 1F).

### Activation of Cre recombinase in Arsb^COIN/COIN^ mice restores ARSB expression.

Since *Arsb^COIN/COIN^* mice replicated many of the hallmarks of MPS VI (11, 14, 15), we explored whether rescue of key phenotypes is possible when *Arsb* is restored at different time points, by delivering tamoxifen to cohorts at juvenile (P7), early adolescence (P21), or adult (P56–P70) stage. Each staged cohort was split into 2 groups — one used to assess disease at 1 month after tamoxifen delivery and the other to assess disease at 3 months after tamoxifen delivery.

**Figure 2:**
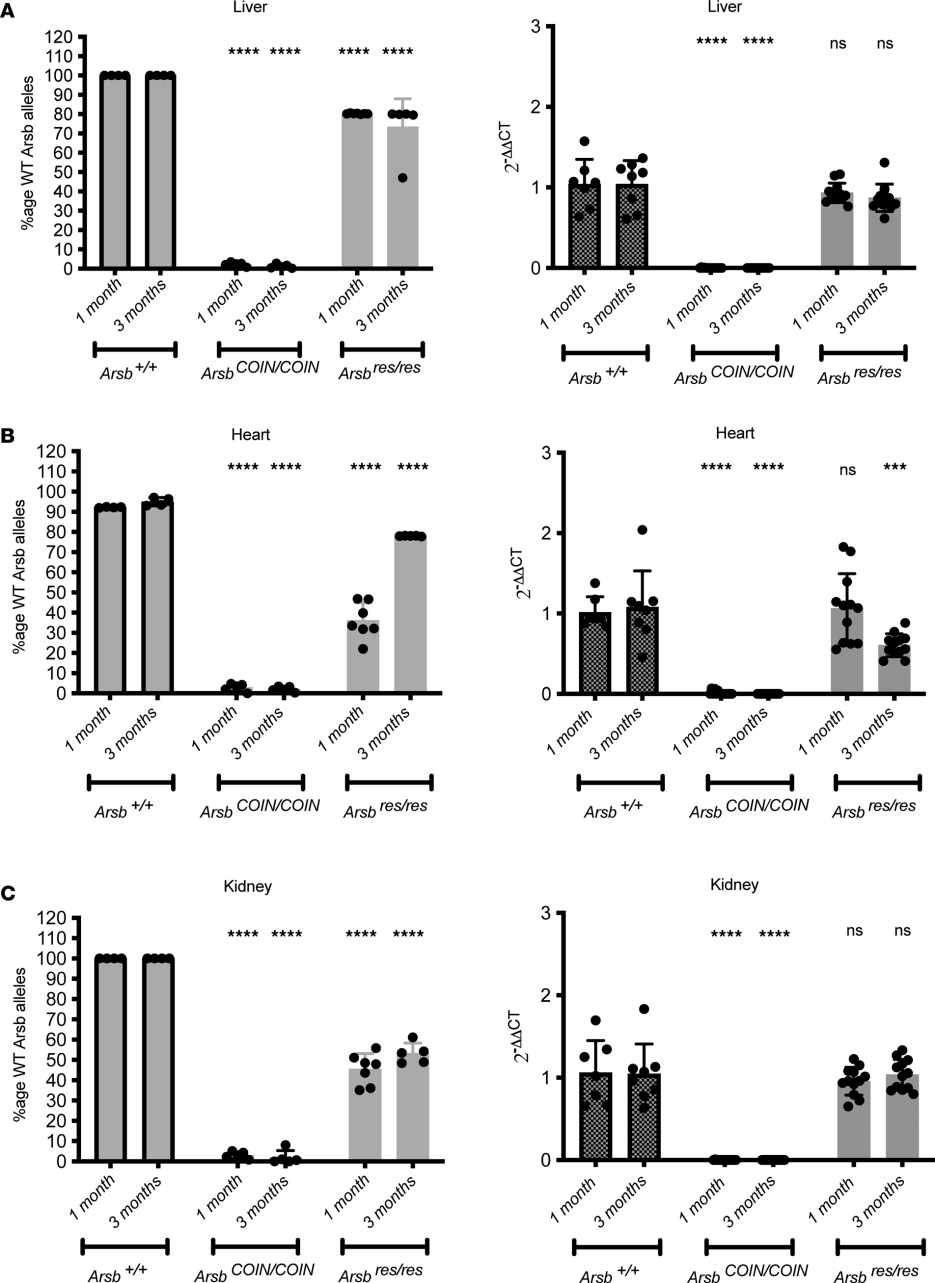
Arsb COIN allele recombination rates and transcript abundance in peripheral organs when tamoxifen is delivered at P7. (**A**–**C**) Percentage recombination of the liver, heart, and kidney is assessed as ΔCT of Arsb Lox71–specific sequence compared with a serial standard, with GAPDH as a reference. Transcript abundance is assessed as ΔΔCT against WT samples as references. *n* = 4–17 mice. Data represent mean ± SD. Statistical significance was determined by 2-way ANOVA with Tukey’s multiple-comparison test. ***P < 0.001, ****P < 0.0001.

We first confirmed whether *Cre* recombinase mediated restoration of *Arsb* exon 5 to the sense strand occurred, by analyzing genomic DNA extracted from peripheral organs. Restoration was observed in 60%–80% of genomic DNA from liver and 30%–60% of genomic DNA from heart and kidney (Figure 2 and Supplemental Figure 2, A–F). Interestingly, there was a significant increase in restored *Arsb* in cardiac genomic DNA at 3 months after tamoxifen delivery in the P7 cohort; 1 month after tamoxifen delivery, the mean was at 36%, whereas at 3 months after tamoxifen delivery, the mean was up to 78%, suggesting a possible competitive advantage for cells with restored *Arsb*. Restoration was not detected in *Arsb^COIN/COIN^* mouse tissues, as would be expected, given that they were not exposed to Cre.

To confirm that restoration of exon 5 to the sense strand via this method results in a functionally WT *Arsb*, we performed quantitative PCR (qPCR) to check for presence of *Arsb* mRNA. Transcript abundance in livers was rescued to WT levels by 3 months after tamoxifen delivery, regardless of at which age the restoration of *Arsb* was initiated (Figure 2 and Supplemental Figure 2). Transcript levels in the heart and kidney ranged from 20% to 70% of WT when restoration occurred at P56–P70 or P21, whereas restoration at P7 resulted in rescue of *Arsb* mRNA to nearly WT levels. No *Arsb* mRNA was detected in RNA extracted from *Arsb^COIN/COIN^* mice lacking Cre, where exon 5 remains inverted (Figure 2 and Supplemental Figure 2, A–F).

### Restoration of Arsb results in normalization of GAG levels.

**Figure 3:**
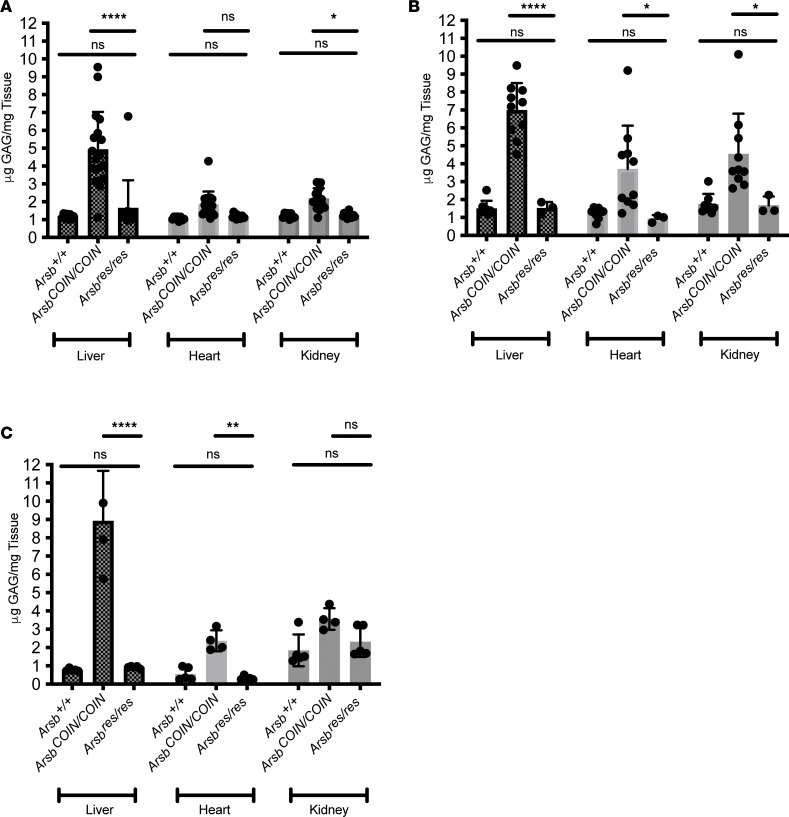
Restoration of Arsb expression normalizes GAG levels in peripheral organs. (**A**–**C**) GAG accumulation is abrogated to WT levels with restoration to WT of the conditional Arsb allele at all ages in peripheral organs 3 months after tamoxifen delivery at P7 (**A**); at P21 (**B**), and at P56–P70 (**C**). *n* = 3–18 mice. Data represent mean ± SD. Statistical significance was determined by 2-way ANOVA with Tukey’s multiple comparisons were performed, as indicated. *P < 0.05, **P < 0.01, ****P < 0.0001.

To further confirm disease-relevant restoration of *Arsb* function, we measured GAG concentration in liver, heart, and kidney. At 3 months after tamoxifen injection, restoration of *Arsb* at all ages was sufficient to reduce GAG accumulation down to WT levels (Figure 3). Similar abrogation of GAG storage was observed at 1 month after tamoxifen injection (Supplemental Figure 3, A–C).

### Restoration of Arsb at P7 rescues tibia lengths.

**Figure 4:**
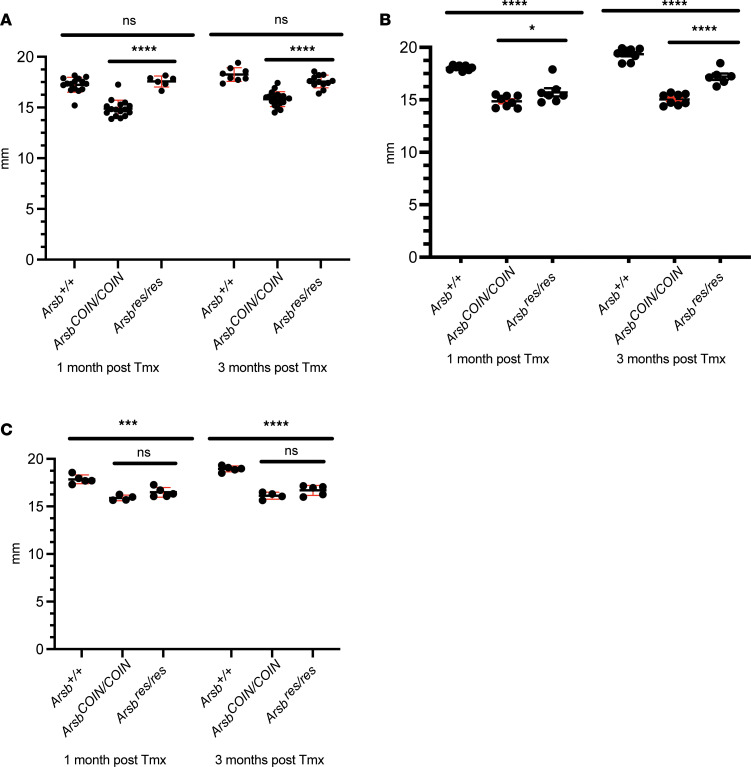
Tibia lengths show little to modest improvement with inversion to WT of the conditional Arsb allele at P56–P70 and P21 but with full rescue to WT levels at P7. (**A**) Tamoxifen was delivered at P7. (**B**) Tamoxifen was delivered at P21. (**C**) Tamoxifen was delivered at P56–P70. All images and quantification were assessed 1 and 3 months after tamoxifen delivery. Statistics were determined by 2-way ANOVA with Tukey’s multiple-comparison test. *P < 0.05, ***P < 0.001, ****P < 0.0001.

To determine whether restoration of *Arsb* to the WT state can rescue skeletal growth, we utilized left tibia length as a surrogate measurement. When *Arsb* was restored starting at P7, there was full correction of the skeletal length phenotype to WT levels at both 1 and 3 months after treatment, whereas the diseased, Cre^–^ group tibia lengths remained significantly shorter (Figure 4A). When *Arsb* was restored starting at P21, tibial lengths showed significant improvement by 1 month after tamoxifen and showed further improvement by 3 months, yet they failed to reach WT lengths (Figure 4B). In the P56–P70 cohort, *Arsb^COIN/COIN^* mice exhibited significantly shorter tibia lengths, which were not corrected at either 1 month or 3 months after tamoxifen treatment in *Arsb^res/res^* mice, where *Arsb* had been restored (Figure 4C).

To further understand the efficacy of *Arsb* restoration at different time points, we determined vertebral column length and cranial length/width ratios using μCT data. When restoration was initiated at P7, there was full rescue to WT lengths by 3 months (Supplemental Figure 4A). When restoration was initiated at P21, there was an increasingly significant difference in L2–L6 lengths from 1 to 3 months after treatment. However, even at 3 months after restoration, vertebral column length failed to reach WT levels (Supplemental Figure 4B). In the P56–P70 mice, there is a moderate, progressive shortening of the vertebral column in the diseased cohort. This is not corrected by 1 month after treatment in the restoration group, although some improvement was observed by 3 months after treatment (Supplemental Figure 4C).

Mirroring the correction observed in the growth of the postcranial skeleton, restoration of *Arsb* at P7 results in normal cranial length/width ratios (Supplemental Figure 5, A and D). In contrast, cranial length/width ratio — a commonly used method to quantify changes in skull and facial morphology — also displayed age-dependent disease manifestation and showed little to modest improvements with restoration of *Arsb* at P21 and P56–P70 (Supplemental Figure 5, B and C). Changes in both vertebral column length and cranial length/width ratios plateaued at lower than WT levels by 3 months after tamoxifen treatment, with no additional improvement seen up to 5 months later (data not shown).

### Restoration of Arsb at P7 rescues tibia width.

In order to assess whether correction of growth plate widths can be achieved when Arsb is restored to WT, quantitative analyses of growth plate widths were performed across groups.

**Figure 5:**
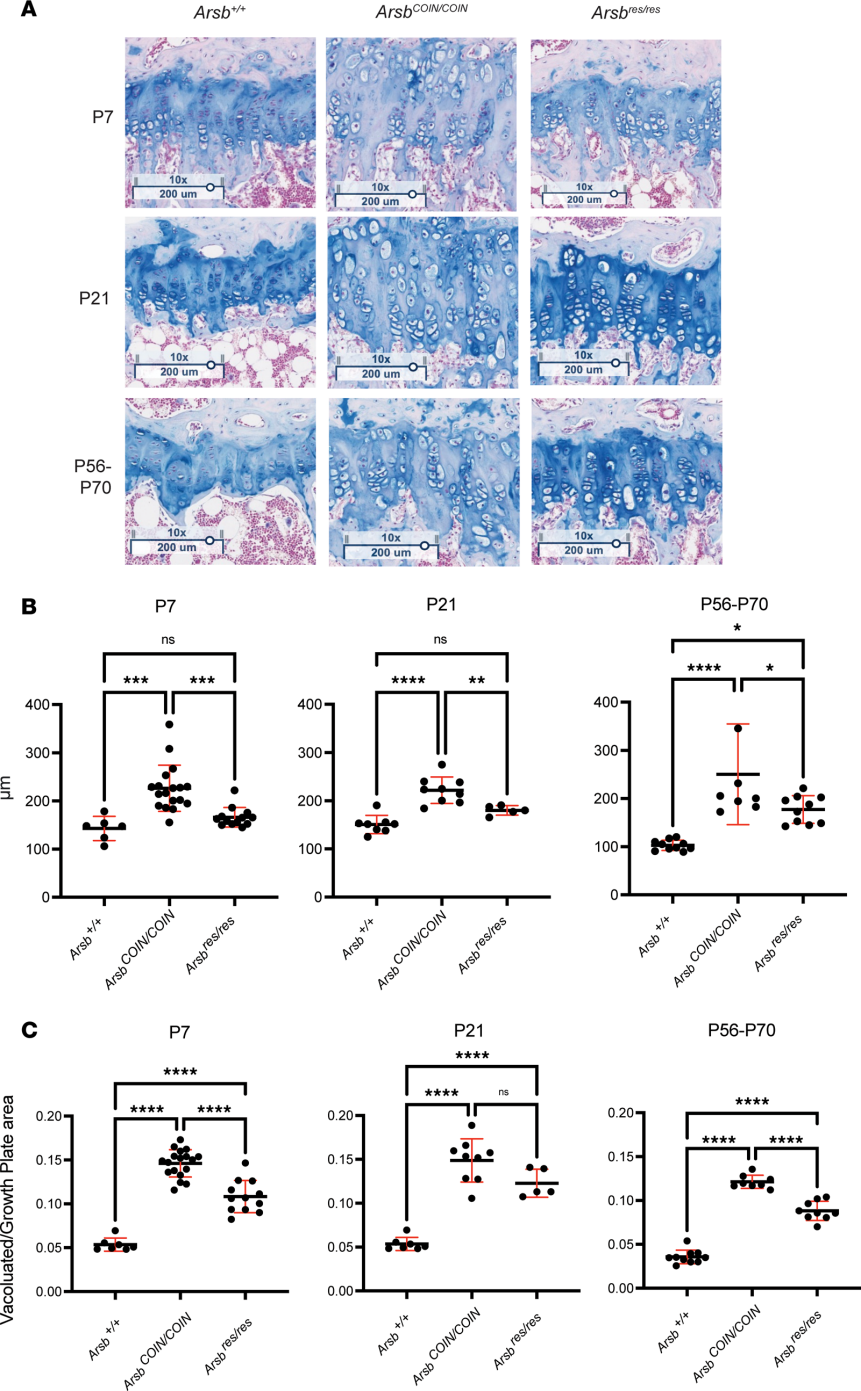
Restoration of Arsb at P7 results in rescue of growth plate defects. (**A**) Alcian blue–stained tibial growth plates from *Arsb^+/+^, Arsb^COIN/COIN^*, and *Arsb^res/res^* mice at 3 months after tamoxifen treatment from P7 (top), P21 (middle), and P56–P70 (bottom) mice. Scale bar: 200 um. (**B**) Quantification of growth plate widths from tamoxifen-treated mice at 3 months after tamoxifen treatment. (**C**) Quantification of growth plate vacuolation from tamoxifen-treated mice at 3 months after tamoxifen treatment. Statistics were determined by 2-way ANOVA with Tukey’s multiple-comparison test. *P < 0.05, **P < 0.01, ***P < 0.001, ****P < 0.0001.

Tibial growth plates from P7, P21, and P56–P70 tamoxifen-treated cohorts were sectioned and processed with the GAG-specific Alcian blue stain at 3 months after treatment (Figure 5A and Supplemental Figure 6). Significant reduction in growth plate width to WT levels is seen with restoration at P7 and P21, with P7 levels much closer to WT levels (Figure 5, A and B). A significant but modest reduction in growth plate width was observed with restoration at P56–P70 (Figure 5, A and B). Interestingly, vacuolation in the growth plate was not fully corrected when restoration was initiated at any age (Figure 5C). Total vacuolated area normalized to growth plate area was reduced at all ages in the restoration group — significantly at P7 and in adulthood — but at no age does it approach WT levels (Figure 5C).

### Restoration of Arsb improves femoral bone mass parameters at all ages.

**Figure 6:**
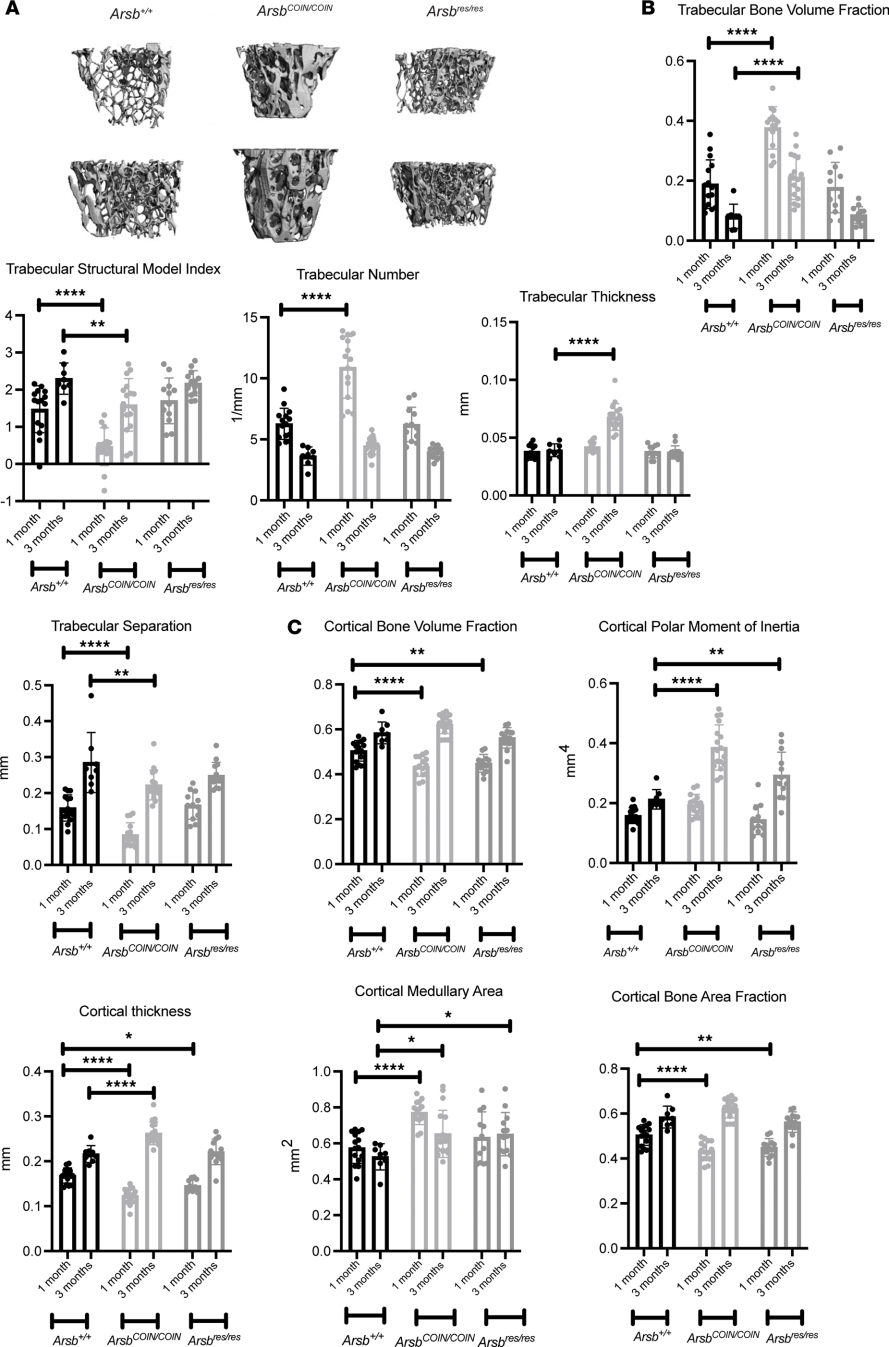
Restoration of Arsb at P7 improves femoral bone mass. (**A**) High-resolution μCT images of femoral distal metaphyses at 3 months after tamoxifen treatment. (**B**) Quantification of trabecular readouts. (**C**) Quantification of cortical readouts. Data are shown as the mean ± SD (*n* = 8–17). Repeated-measures 1-way ANOVA was performed, as indicated. *P < 0.05, **P < 0.01, ****P < 0.0001.

Trabecular bone, located subproximal to growth plates in long bones, is substantially remodeled after skeletal patterning has been completed. Since *Arsb* homozygous-null mice display altered trabecular architecture (11, 14, 21), we explored whether our model displayed similar defects and whether they could be prevented or corrected by restoration of *Arsb*. To explore trabecular architecture of long bones, we utilized high-resolution ex vivo μCT of left femurs from P7 and P56–P70 tamoxifen-treated cohorts. Consistent with previous models, *Arsb^COIN/COIN^* mice displayed increased density in the trabecular space (Figure 6A), with significantly increased trabecular bone volume, increased number of trabeculae, increased trabecular thickness, and decreased separation (Figure 6B). These parameters correlate with a decrease in structural model indices. Midshaft, mature cortical bone displays a similar phenotype, with significantly increased cortical bone volume, cortical thickness, and total cortical bone area fraction (Figure 6C). Cortical parameters also correlated with differences in shape parameters— e.g., the increase observed in cortical polar moment of inertia. Restoration of *Arsb* at P7 rescued these phenotypes and restored trabecular bone parameters to WT levels (Figure 6, A–C), consistent with previous reports (14). Analyses performed in cohorts where rescue was initiated at P56–P70 (Supplemental Figure 7) exhibited similar trends, although the differences were not statistically significant. 

### Genetic restoration of Arsb is more efficacious than galsulfase ERT.

**Figure 7:**
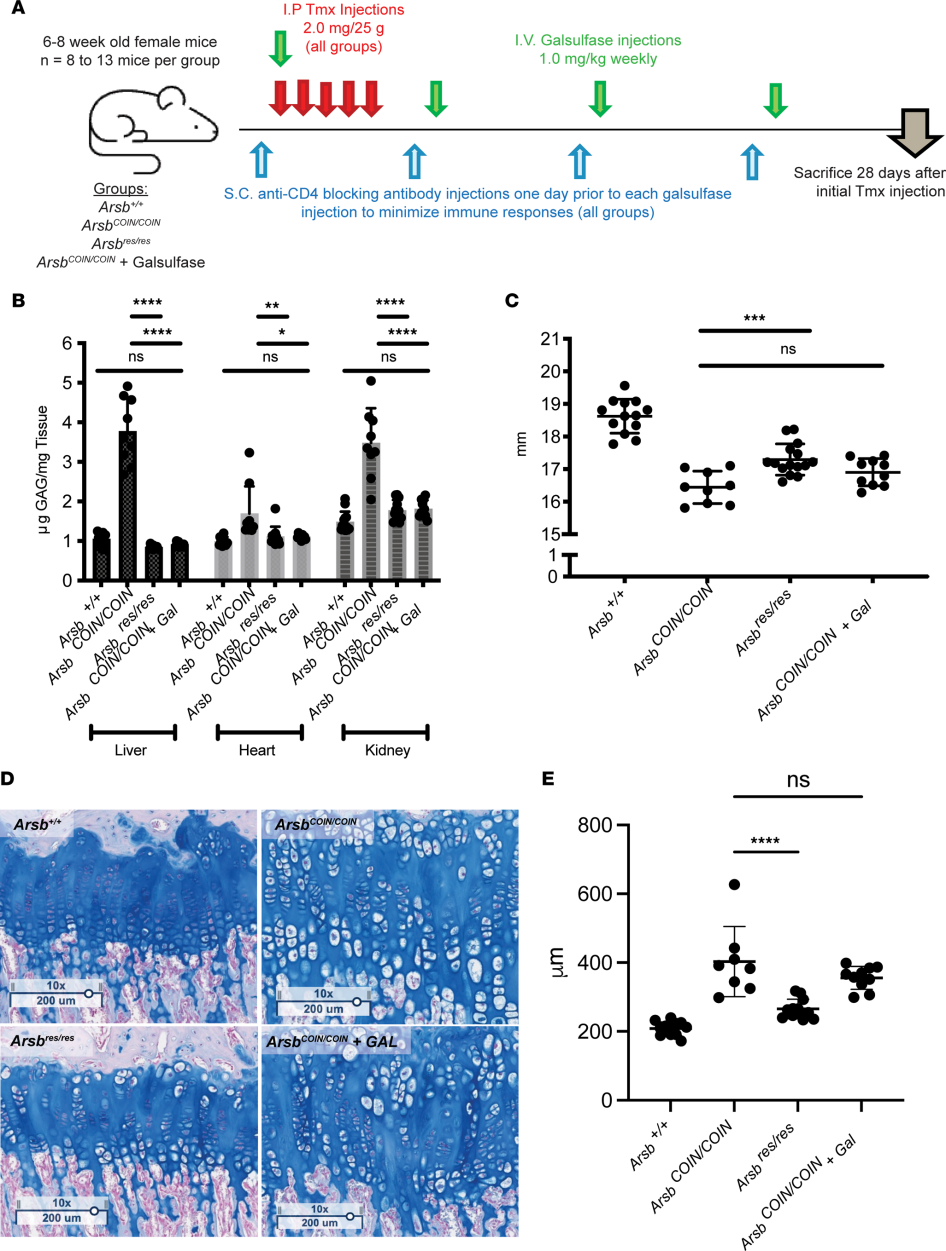
Restoration of Arsb expression improves skeletal phenotype better than enzyme replacement therapy. (**A**) Experimental design. (**B**) GAG accumulation in liver, heart, and kidney. (**C**) Left tibia lengths. (**D**) Alcian blue stain of tibial growth plates. Scale bar: 200 um. (**E**) Quantification of growth plate widths. Two-way ANOVA with Tukey’s multiple-comparison test were performed, as indicated. *P < 0.05, **P < 0.01, ***P < 0.001, ****P < 0.0001.

In order to discern to what degree the current therapeutic standard of care addresses MPS VI phenotypes, we directly compared galsulfase ERT in *Arsb^COIN/COIN^* mice with restoration of *Arsb* to the WT state. WT and untreated *Arsb^COIN/COIN^* mice were used as additional control groups (Figure 7A). For this comparison, we chose to use young adult mice instead of neonates in order to accommodate the required number of CT scans and drug injections and to provide clearer readouts, since the phenotype of MPS VI is more profound with age. As with prior experiments, all groups received tamoxifen in order to control for drug-specific effects. Additionally, a CD4 blocking antibody was delivered weekly to all groups 1 day before each galsulfase delivery, in order to minimize immune response to galsulfase ERT (Figure 7A). All mice were μCT scanned at 26 days and euthanized 28 days after the first tamoxifen injection.

Quantification of GAG abundance in the liver, heart, and kidney showed robust depletion of total GAG content in both the *Arsb^res/res^* and galsulfase treated groups, while the untreated *Arsb^COIN/COIN^* control group showed significantly elevated levels (Figure 7B). Left tibia lengths were partially rescued in the *Arsb^res/res^* group, while the galsulfase-treated group showed no significant difference from untreated *Arsb^COIN/COIN^* mice (Figure 7C). Tibial growth plates widths followed a similar trend — the *Arsb^res/res^* restoration group showed condensation of growth plate widths, which were profoundly different from *Arsb^COIN/COIN^* — while the galsulfase-treated group showed no differences (Figure 7, D and E).

## Discussion

MPS manifestations include a spectrum of dysostosis multiplex, also known as progressive skeletal dysplasia (1–3). Although the advent of ERT for a subset of these disorders has resulted in some progress in their management, it has had only marginal efficacy in treating the skeletal manifestations. The inability of ERT to treat skeletal manifestations is apparent in MPS VI, where galsufase ERT is successful in treating soft tissue manifestations yet achieves limited efficacy in correcting the skeletal phenotypes, with a sporadically reported, modest improvement in shoulder flexion (2, 4, 11). Two factors may be contributing to this marginal efficacy: inability of the enzyme to reach need-to-treat tissues and irreversibility of the skeletal phenotypes. In order to address the latter possibility, and to explore how timing of treatment during postnatal skeletal development affects efficacy, we generated a new mouse model of MPS VI where function of Arsb can be restored at desired time points. This model is based on COIN methodology (17), wherein the *Arsb* gene starts as null and can be restored using Cre. Since *Arsb^COIN^* does not rely on exogenous delivery of ARSB (akin to galsufase ERT), it mitigates the variables associated with ERT access to need-to-treat tissues and allows for the focus to move specifically to the question of reversibility and timing.

*Arsb^COIN/COIN^* mice replicate the main clinical features of MPS VI and phenotypically match other MPS VI mouse models (11, 14, 15). For example, *Arsb^COIN/COIN^* mice display elevated GAG storage in liver, heart, and kidney, along with multiple phenotypes in the skeletal system. Dysostosis multiplex is modeled in *Arsb^COIN/COIN^* mice, observed as shortened tibial length, vertebral column compression, decreased cranial length/width ratios, tibial growth plate widening, and markedly increased vacuolation of growth plate cells.

In order to explore whether restoration of Arsb can rescue the phenotypes of *Arsb^COIN/COIN^* mice, we introduced a globally expressed tamoxifen-regulated Cre driver, *Gt(ROSA26)Sor^CreERt2^*. Activation of Cre using tamoxifen results in conversion of *Arsb^COIN^* allele to WT, *Arsb^res^*. In this manuscript, we use *Arsb^COIN/COIN^* to refer to mice without a *Cre* allele and *Arsb^res/res^* to refer to mice with active *Cre* induced by tamoxifen treatment. By effecting restoration of *Arsb* at different times during postnatal development, we demonstrate that early treatment (i.e., by tamoxifen-induced restoration of *Arsb* initiated at P7) is much more effective in correcting the skeletal manifestations of MPS VI than treatment at later time points (P21 and P56–P70). To our knowledge, *Arsb^COIN/COIN^*; *Gt(ROSA26)Sor^CreERt2/+^* mice are the first model of MPS VI in which near-complete correction of the skeletal phenotypes has been demonstrated. Not surprisingly, in *Arsb^COIN/COIN^*; *Gt(ROSA26)Sor^CreERt2/+^* mice treated with tamoxifen at P21, only partial rescue is possible, while mice treated at P56–P70 are past the window for major bone growth and exhibit no discernible improvement.

While most skeletal disease parameters seem to be restored to WT levels, increased vacuolation in the growth plate is not completely rescued at any age. It is unclear whether this is due to partial allele conversion rate in the growth plate or an aspect of disease activity that cannot be rectified. The vacuolation phenotype may also be due to relative lack of metabolic or transcriptional activity of certain cell types, on which *Arsb* restoration may have little impact (22, 23).

We further used this new model to explore the effectiveness of galsufase ERT, initiating treatment in P21 *Arsb^COIN/COIN^*; *Gt(ROSA26)Sor^CreERt2/+^*, and comparing the efficacy of galsulfase ERT to genetic restoration of *Arsb*. Although galsulfase ERT was efficacious in normalizing GAG levels in peripheral tissues, it did not rescue the skeletal phenotypes. This was in contrast to the partial yet sizeable rescue obtained with genetic restoration of *Arsb*.

Taken together, these results indicate that the skeletal phenotypes of MPS VI are indeed reversible, and they further suggest that exogenous enzyme may be limited in efficacy due to inability to reach the growth plates (7). Our results also imply that, if delivered early enough, an optimized therapeutic should be able to treat both stature and dysmorphia-related symptoms of MPS VI. It is important to note that mouse skeletal developmental age correlations with human developmental age are varied; nonetheless, if we accept that P7 in mice is comparable with the first few years of human development (24), then our data suggest that rescue of the skeletal phenotypes should be possible if treatment occurs within the first few years of life.

## Methods

### Mouse lines and injections

#### Mouse line generation.

A large targeting vector (LTVEC) with a 5′ homology arm comprising 81 kb of the mouse *Arsb* locus and 3′ homology arm comprising 102 kb of the mouse *Arsb* locus was generated to replace a region surrounding exon 5 (ENSMUSE00001413545, Ensembl release 109) with a corresponding inverted region flanked by a 5′ lox71 site and a 3′ lox66. A roxed self-deleting cassette was inserted downstream of the inverted sequence. To generate the mutant allele, the LTVEC was introduced into mouse embryonic stem cells. Specifically, 2 × 10^6^ mouse VGF1 embryonic cells (50% C57BL/6NTac 50% 129S6/SvEvTac) carrying CreER^T2^ in the *Gt(ROSA26)Sor* locus (17) were electroporated with 0.4 μg mArsb LTVEC. Antibiotic selection was performed using G418 at a concentration of 100 mg/mL. Colonies were picked, expanded, and screened by TaqMan. F0 mice were generated from above modified ES cells using the VelociMouse method. Specifically, mouse ES cell clones described above were selected and injected into 8 cell-stage embryos using the VelociMouse method (25–27).

For the initial experiment to assess MPS VI–like phenotypes in *Arsb^COIN/COIN^* mice, mice were anesthetized with isoflurane at 18–19 weeks of age prior to μCT scans. Two days later, they were CO_2_ euthanized, and liver, heart, spinal cord, spleen, kidney, and sera were harvested.

#### Injections.

For P7, P21, and P56–P70 restoration experiments, tamoxifen (MilliporeSigma, T5648) was resuspended in sterile corn oil (MilliporeSigma, C8267) at 10 mg/mL or 5 mg/mL and injected via the i.p. route at the appropriate doses normalized to mouse weight daily for 5 consecutive days. For direct comparison with ERT, T cell depletion was accomplished with i.p. injection of an α-CD4 antibody (BioXCell, BE0003-3) at 50 mg/kg, weekly, starting at the day –1. Galsulfase delivery was accomplished with retro-orbital i.v. injection of recombinant human ARSB (BioMarin, Naglazyme lot L061938) at 1 mg/kg, weekly, starting at day 0. Tamoxifen injections were also started at day 0, injected via the i.p. route at 2.0 mg/25 grams mouse weight for 5 consecutive days. Antibodies and enzymes were diluted to appropriate doses in 0.9% sodium chloride (Intermountain Life Sciences, Z1376).

### Recombination assays

Genomic DNA were extracted from liver, heart, kidney, and spleen tissue harvested from MPS VI COIN and control mice using the GenElute Mammalian Genomic DNA Miniprep Kit (Sigma-Aldrich, G1N350-1KT). Genomic DNA from untreated COIN and WT mice were mixed at serial percentages to produce standard curves (100%, 90%, 75%, 60%, 40%, 20%, and 0% COIN genomic DNA) on each plate. In total, 50–100 ng of genomic DNA were used per reaction in qPCR using the 2× PowerUp SYBR Green Master Mix (Thermo Fisher Scientific, A25742) with 1.0 μM of the following primers as appropriate: EHW092 (Arsb COIN fwd [5′–3′]): AGGCCAAGATTGACAGTTACCAG; EHW093 (Arsb COIN rev [5′–3′]): GGAGTACAGGGAAGGAAACCT; EHW094 (GAPDH fwd): CATGGCCTTCCGTGTTCCTA; EHW095 (GAPDH rev): CCTGCTTCACCACCTTCTTGAT. Technical duplicates for GAPDH reactions and technical triplicates for Arsb COIN reactions were used to assess average CT values. ΔCT values were compared with standards to assess percentage recombination.

### qPCR

#### For RNA extraction.

Tissue samples were homogenized in TRIzol, and chloroform was used for phase separation. The aqueous phase, containing total RNA, was purified using MagMAX-96 for Microarrays Total RNA Isolation Kit (Ambion, Life Technologies) according to manufacturer specifications. Genomic DNA was removed using RNase-Free DNase Set (Qiagen).

#### First-strand synthesis and qPCR.

mRNA was reverse transcribed into cDNA using SuperScript VILO Master Mix (Invitrogen, Life Technologies). cDNA was amplified with the SensiFAST Probe Lo-ROX (Meridian) using the 12K Flex System (Applied Biosystems). GAPDH was used to normalize any cDNA input differences. Primer sequences used were the following: Arsb fwd: CCAAACCTCTGGATGGCTTCAAC; Arsb rev: GTCCTGATCGATGTTGTGTAGCAG; Arsb probe: AAGACAATCAGTGAAGGACACCCATCCC; Gapdh fwd: TGCCCAGAACATCATCCCT; Gapdh rev: GGAGGCCATGCCAGTGAG; Gapdh probe: ATCCACTGGTGCTGCCAAGGCTG. Data were acquired in technical triplicate and were calculated with mean CT using the ΔΔCT method, with WT samples as references.

### GAG accumulation assays

#### Tissue digestion for GAG isolation.

Finely minced organ tissue was resuspended at a concentration of 10 mg/mL in 0.3 mg/mL papain + 2 mM DTT and incubated at 60°C for 1.5 hours, with samples gently vortexed every 15 minutes. In total, 10 μL of 1M acetic acid and 40 μL of Tris-HCl (pH 8.0) were added per 1 mL of organ lysate and mixed well (AMSBio, 280560-TDK).

#### Sulfated GAG quantitation.

A standard curve was produced using five 4-fold serial dilutions from 100 μg/mL chondroitin sulfate. Four-fold dilutions of each sample was created in duplicate in 1× assay buffer (AMSBio, 280560-N). In total, 100 μL of standards and experimental samples were transferred to a clear-bottom plate and then mixed with 100 μL of 100 mM DMMB. Absorbances at 515 nm were assessed within 15 minutes and compared with standard curves (AMSBio, 280560-N).

### Skeletal analyses

For μCT analyses, mice were anesthetized using isoflurane and dynamically scanned using 17 seconds × 2 or 8 seconds × 3 algorithms on Quantum FX or Quantum GX computerized tomographs (PerkinElmer) to produce whole-skeletal images. Stitched vox files were analyzed using Analyze 14.0 software. All lengths were determined using the line-draw tool and double checked with single-blinded analyses. For high-resolution μCT femoral analyses, mice were CO_2_ euthanized, and left femurs were extracted before being drop fixed in 10% formalin (VWR, 16004-121). Samples were incubated rotating at 4°C for 48 hours, washed 4 times with 1× DPBS (Thermo Fisher Scientific, 14040), and stored in 70% ethanol until scans on a Scanco μCT35. Transverse CT slices were evaluated in the region starting 360 μm proximal to the growth plate and extending 1,440 μm. The trabecular bone region was identified manually by tracing the region of interest. Images were thresholded using an adaptive-iterative algorithm, and morphometric variables were computed from the binarized images using direct, 3D techniques that do not rely on any prior assumptions about the underlying structure. Transverse CT slices were also evaluated in the midshaft region, extending 120 μm.

### Histology and growth plate analyses

Right knee joints were harvested from freshly CO_2_-euthanized mice and drop fixed in 4% paraformaldehyde. Samples were fixed for 48 hours, decalcified, embedded in paraffin, and sectioned at 2 levels on the longitudinal axis. Sections were stained with Alcian blue with fast red counterstain, or Safranin O (SafO) with fast green counterstain (Histoserv). Growth plate widths were assessed on Alcian blue–stained samples as a mean with HALO software (Indica Labs), using the layer thickness tool at an approximate interval of 55 μm with maximal smoothing. Vacuolation was assessed on SafO-stained samples with HALO software using the Vacuole Module as total vacuolated area over total growth plate area.

### Statistics

Statistical significance was defined as *P* < 0.05. All experiments were performed with technical duplicates or triplicates and a minimum of biological triplicates. Data analyses were performed using Graphpad Prism software (version 9.0). For comparisons with 2 groups, 2-tailed Welch’s *t* tests were used or 1-way ANOVA with repeated measures. For comparisons with 3 groups, 2-way ANOVA with Tukey’s multiple-comparison test were utilized.

### Study approval

All mouse experiments were approved by the IACUC at Regeneron Pharmaceuticals and were conducted in accordance with the NIH *Guidelines for the Care and Use of Laboratory Animals* (National Academies Press, 2011).

### Data availability

Values for all data points in graphs are reported in the Supporting Data Values file.

## Author contributions

EHW designed and conducted experiments, analyzed data, and wrote the paper. GA, MF, and A Bhargava aided with and/or performed data analysis. ND, XZ, NA, KG, and NR aided with experiments. A Baik, AF, JLS, and KN advised on study design. MM and JR produced and designed recombinant mice, respectively. ANE and KDC conceived of and supervised the study. All authors reviewed the manuscript.

## Supplementary Material

Supplemental data

Supporting data values

## Version 1 09/26/2023

In-Press Preview

## Version 2 11/08/2023

Electronic publication
