# Supplementary material for: Skeletal phenotype amelioration in mucopolysaccharidosis VI requires intervention at the earliest stages of postnatal development
Source: JCI Insight. 2023 Nov 8;8(21):e171312. doi: 10.1172/jci.insight.171312 (PMC10721280; doi:10.1172/jci.insight.171312)
Supplement: Supplemental data [file jciinsight-8-171312-s020.pdf]

2

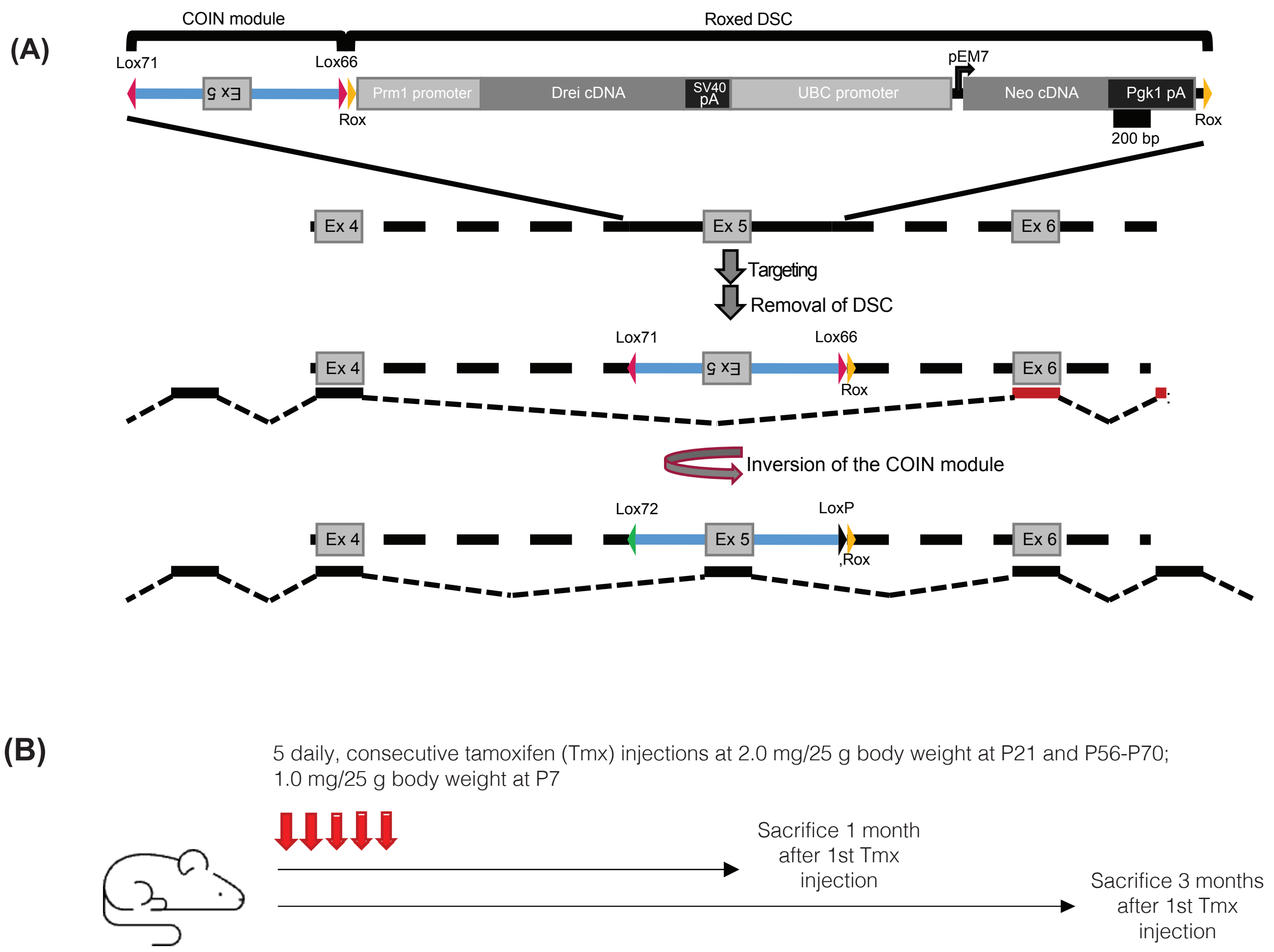

Supplemental Figure 1. Using *Arsb*$^{COIN/COIN}$ mice to model treatment at different timepoints. (A) *Arsb*$^{COIN/COIN}$ allele design **(B)** Experimental setup. COIN = Conditional Inversion, DSC = drug selection cassette

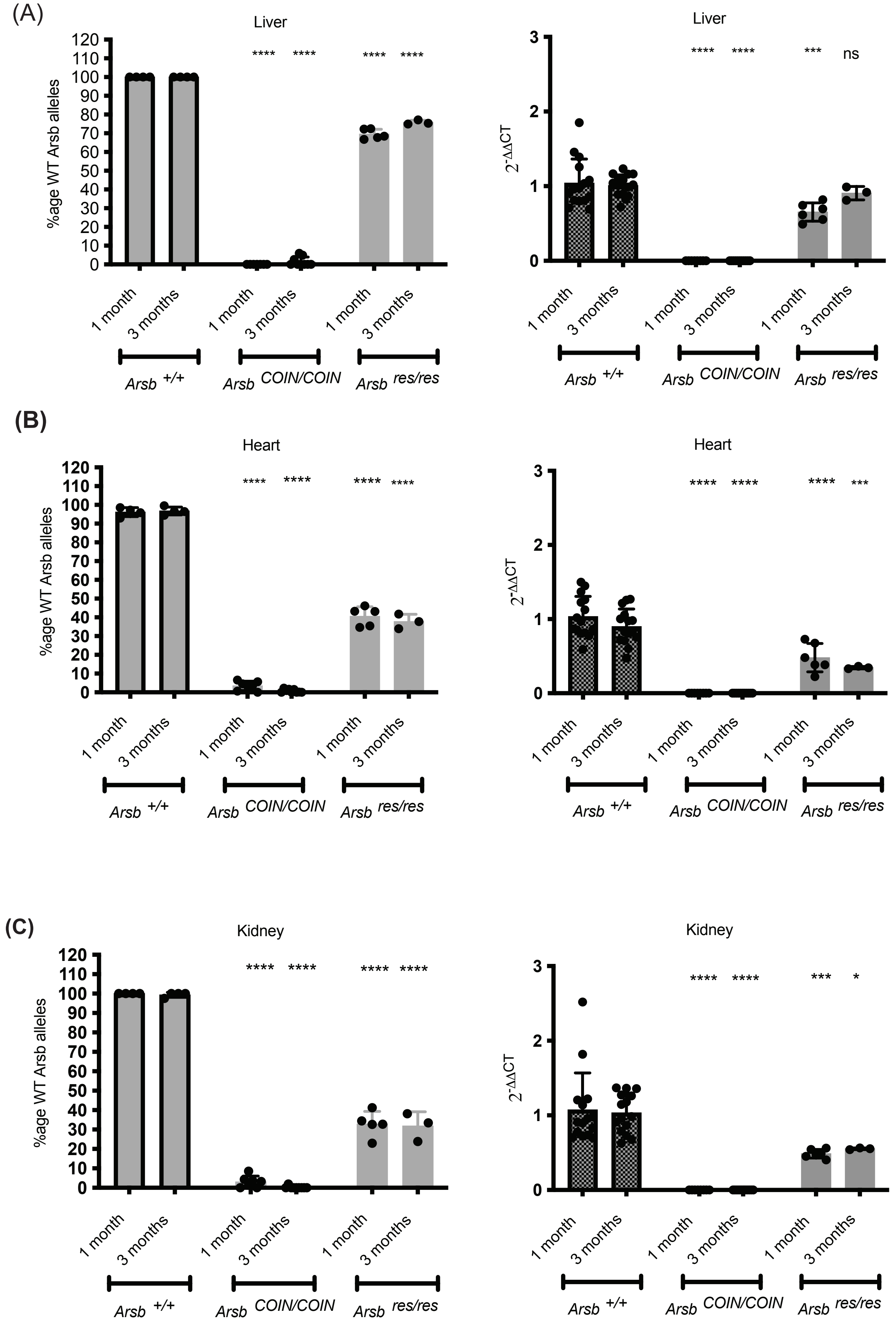

(A)
Liver
%age WT Arsb alleles
2-ΔΔCT
**** **** **** ****
**** **** *** ns
1 month
3 months
Arsb +/+
Arsb COIN/COIN
Arsb res/res
(B)
Heart
**** **** **** ****
**** **** **** ***
(C)
Kidney
**** **** **** ****
**** **** *** *

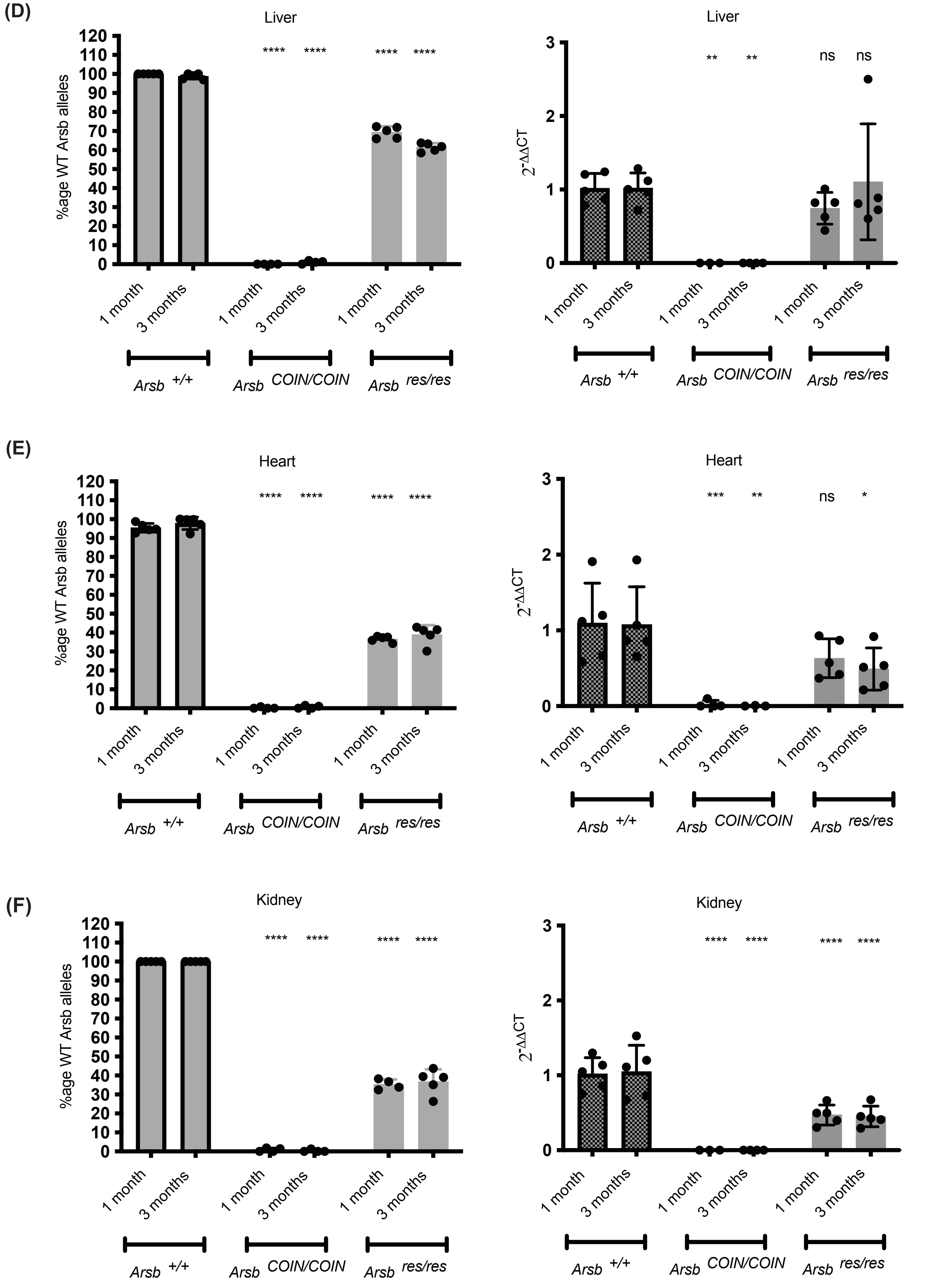

Supplemental Figure 2. $Arsb^{COIN/COIN}$ allele recombination rates and transcript abundance in peripheral organs when tamoxifen is delivered at P21 or P56-P70 show recombination occurs with tamoxifen delivery, and corresponds with appearance of transcript. **(A)** Liver at P21 **(B)** Heart at P21 **(C)** Kidney at P21 **(D)** Liver at P56-P70 **(E)** Heart at P56-P70 **(F)** Kidney at P56-P70. Percentage recombination is assessed as ΔCT of Arsb Lox71 specific sequence compared to a serial standard, with GAPDH as a reference. Transcript abundance is assessed as ΔΔCT against Wt samples as references. Data are shown as the mean +/- SD (n=3 to 9). Two-way ANOVAs with Tukey's multiple comparisons were performed, as indicated. **P*<.05, ***P*<.01, ****P*<.001, *****P*<.0001, ns = not significant

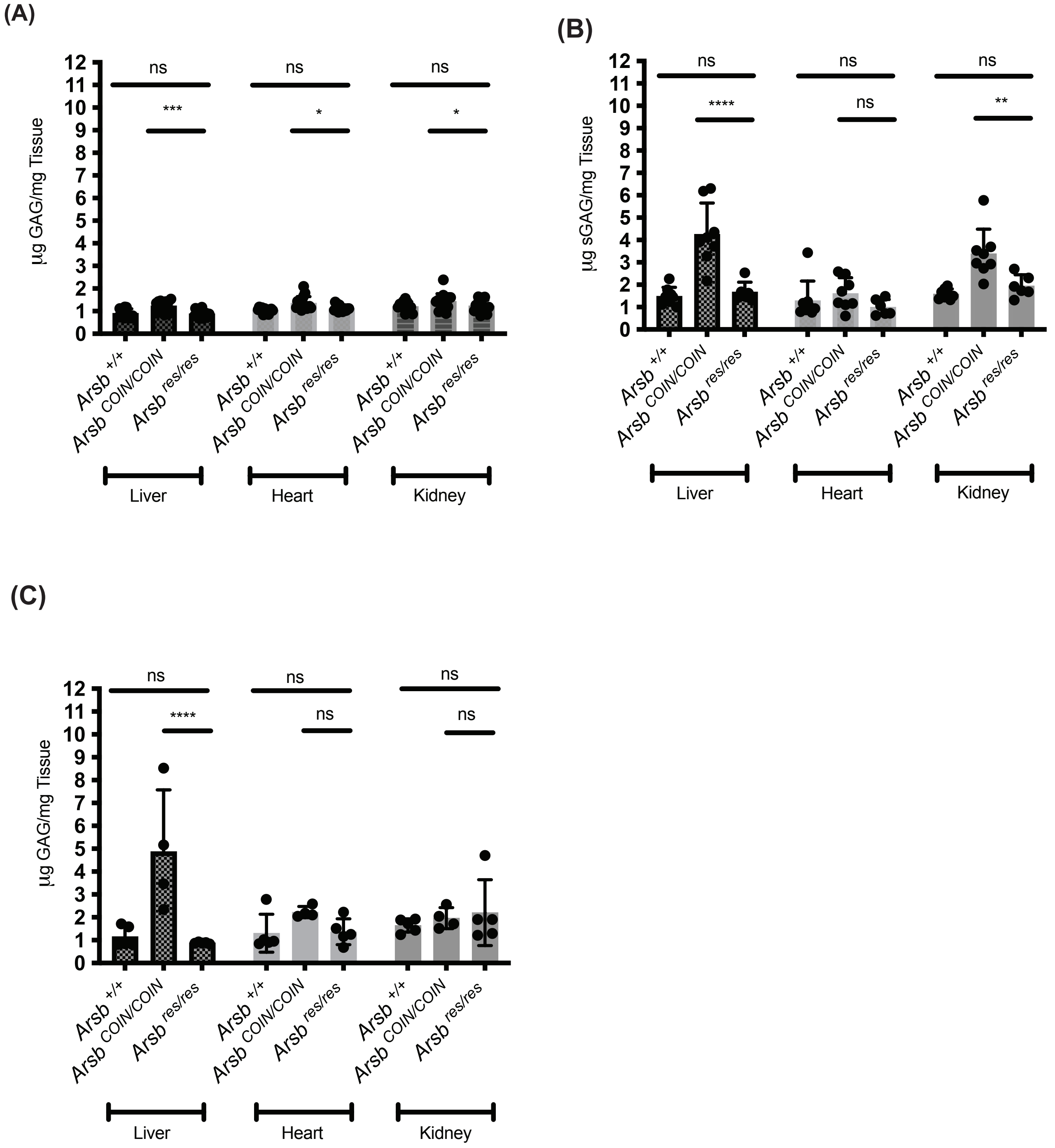

Supplemental Figure 3. Restoration of Arsb expression corrects GAG levels to normal in peripheral organs independent of initiation of treatment. (A) 1 month after tamoxifen is delivered at P7; **(B)** 1 month after tamoxifen is delivered at P21 **(C)** 1 month after tamoxifen is delivered at P56-P70. Data are shown as the mean +/- SD (n =4 to 16). Two-way ANOVAs with Tukey's multiple comparisons were performed, as indicated. **P*<.05, ***P*<.01, ****P*<.001, *****P*<.0001, *ns* = not significant

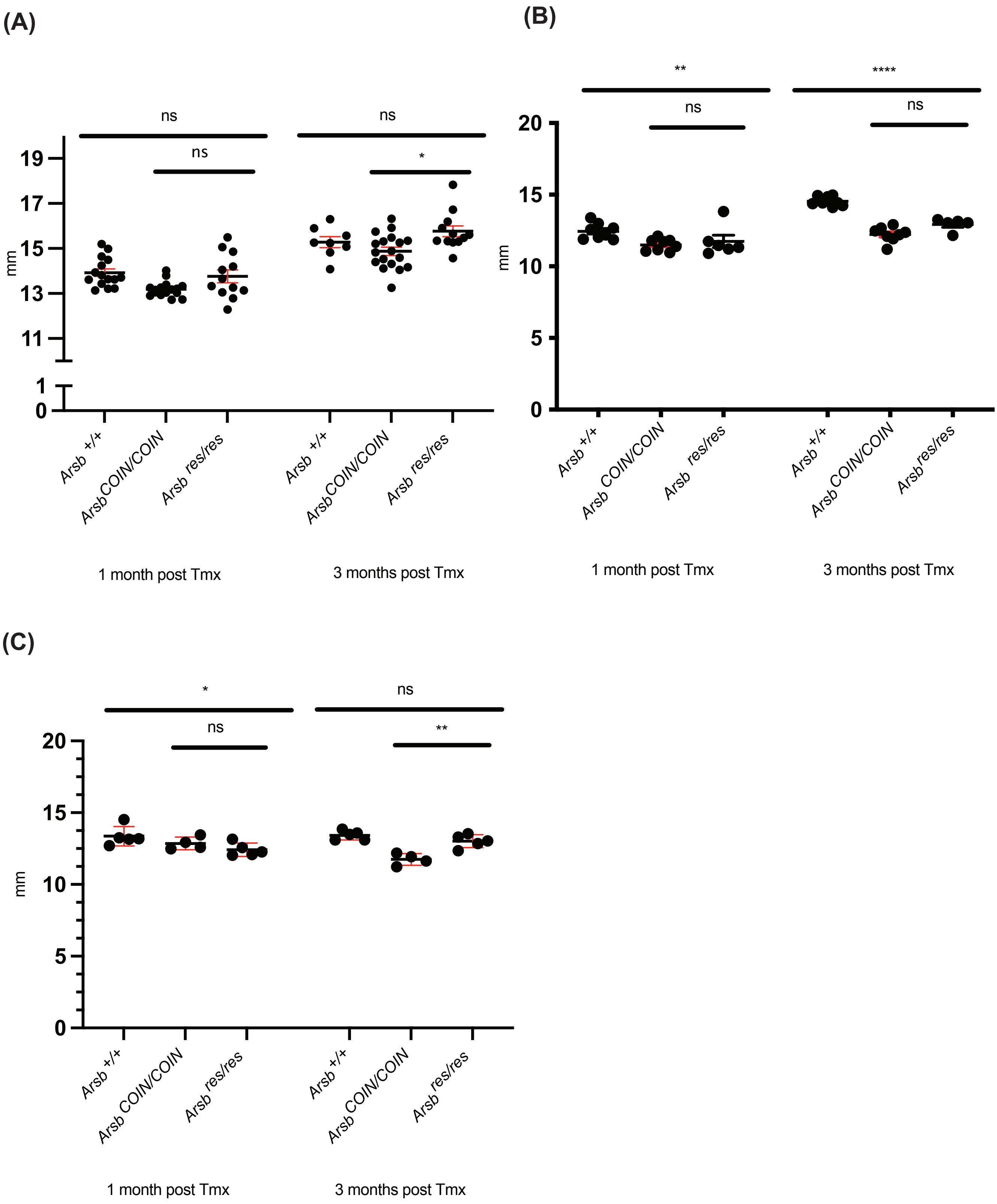

Supplemental Figure 4. Vertebral Column Lengths show full rescue to WT levels with restoration at P7. L2 to L6 lengths were approximated with **(A)** tamoxifen delivered at P7; **(B)** tamoxifen delivered at P21; **(C)** tamoxifen delivered at P56-P70. Two-way ANOVAs with Tukey's multiple comparisons were performed, as indicated. *P<.05, **P<.001, ****P<.0001, *ns* = not significant

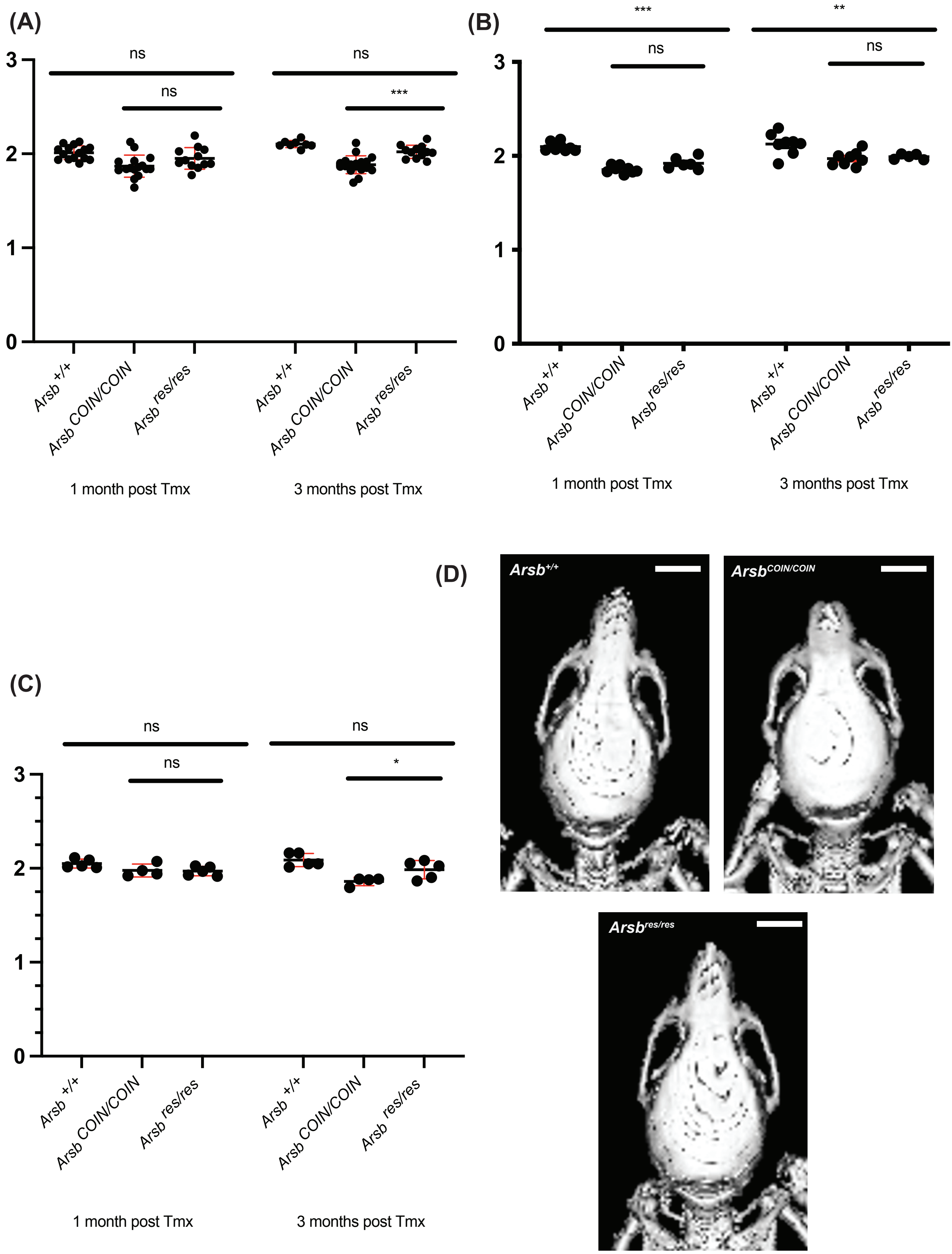

Supplemental Figure 5. Cranial Length to Width Ratios show full rescue to WT levels with Arsb restoration at P7. (A) tamoxifen delivered at P7; **(B)** tamoxifen delivered at P21; **(C)** tamoxifen delivered at P56-P70; **(D)** Cranial radiographs from representative male mice at 3 months post P7 Arsb restoration, dorsal view. Two-way ANOVAs with Tukey's multiple comparisons were performed, as indicated. **P*<.05, ***P*<.01, ****P*<.001, *ns* = not significant

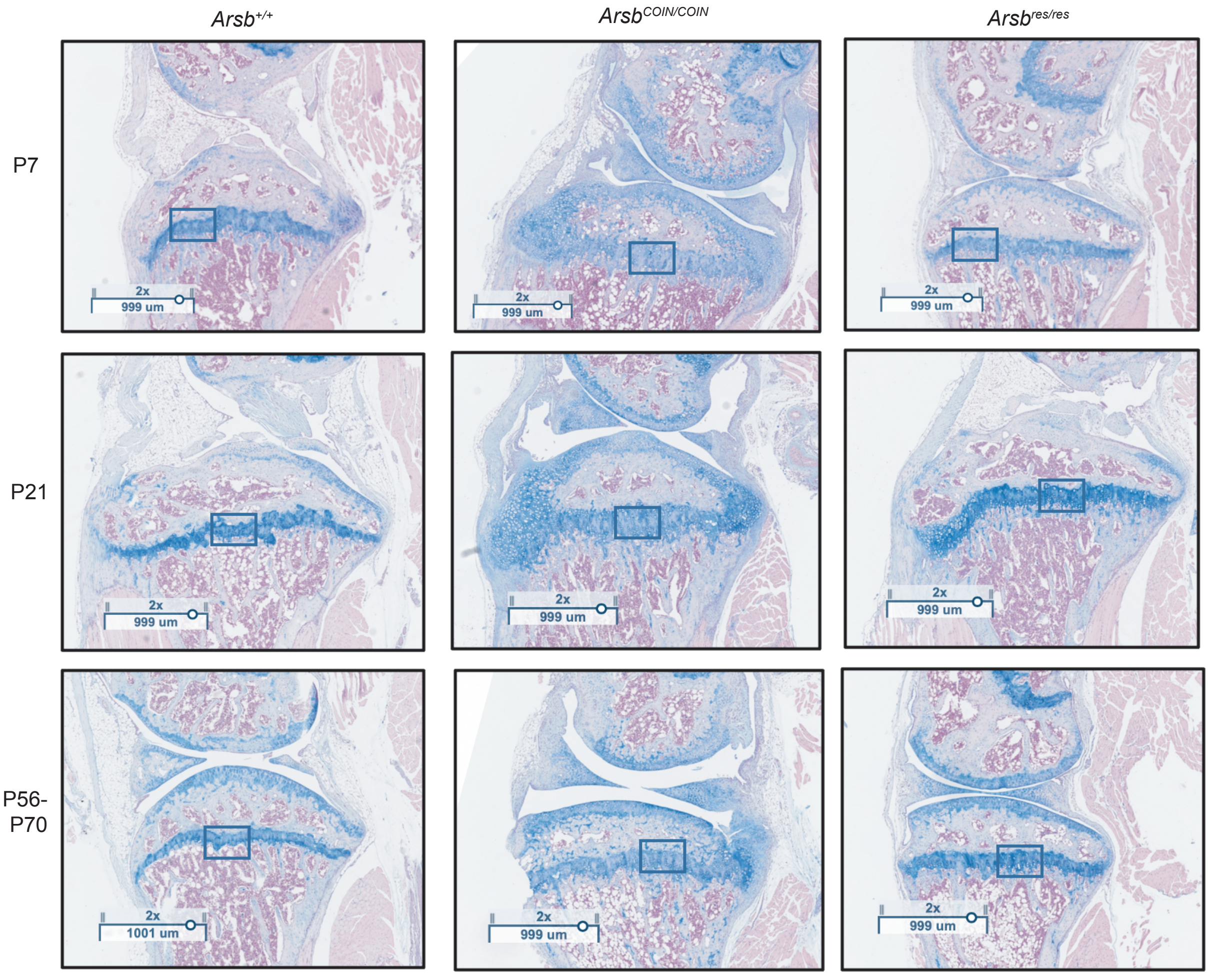

Supplemental Figure 6. Restoration of *Arsb* at P7 results in rescue of growth plate defects. Alcian blue stained tibial growth plates from *Arsb*$^{+/+}$, *Arsb*$^{COIN/COIN}$, and *Arsb*$^{res/res}$ mice at 3 months post tamoxifen treatment from P7 (top), P21 (middle), and P56-P70 (bottom) mice at 2X magnification. Rectangular boxes indicate regions depicted at 10X in Figure 5A.

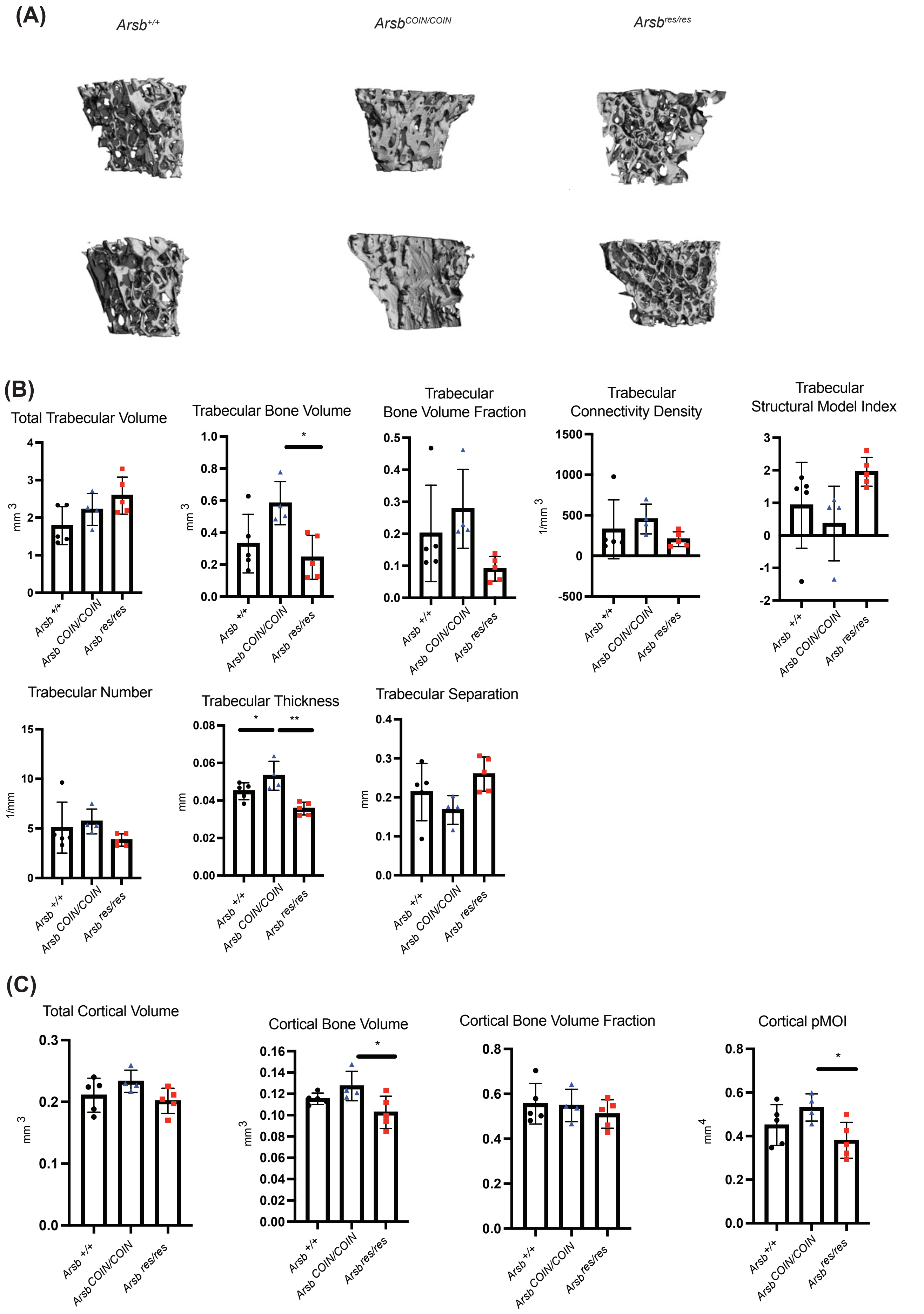

Supplemental Figure 7. Restoration of Arsb expression at P56-P70 improves femoral bone mass. (A) High resolution microCT images of femoral distal metaphyses at 3 months post tamoxifen treatment **(B)** quantification of trabecular readouts **(C)** quantification of cortical readouts. Data are shown as the mean +/- SD (n=4 to 5). Repeated measures one-way ANOVAs were performed, as indicated. *P<.05, **P<.01, ***P<.001, ****P<.0001
